# Is It Time to Give Breast Cancer Patients a Prescription for a Low-Fat Diet?

**DOI:** 10.1093/jncics/pky066

**Published:** 2019-01-07

**Authors:** Jennifer A Ligibel

**Affiliations:** Division of Women’s Cancers, Dana-Farber Cancer Institute, Boston, MA

Observational data consistently link obesity, inactivity, and poor dietary quality to increased risk of developing and dying from malignancy (1–6), but few randomized trials have tested the impact of weight loss, increased exercise, or dietary change on cancer risk or prognosis. The Women’s Health Initiative Low Fat Dietary Modification (WHI DM) Trial is the only fully powered, randomized trial to test the impact of lifestyle change on the risk of developing breast, colorectal, or other cancers (7). The study randomized 48 835 postmenopausal women to a dietary intervention designed to reduce fat intake and increase daily servings of fruits and vegetables, or to a usual diet control group. Although the dietary intervention did not lower the risk of breast, colorectal or total cancers, the trial has provided novel and important information about the relationship between lifestyle factors and a myriad of health outcomes (8–12). 

Two recent publications report on the relationship between group assignment (dietary intervention vs usual care control) and mortality in 1764 women who were diagnosed with breast cancer during their participation in the WHI DM trial (13,14). There was a slight imbalance in the proportion of cancers that were progesterone-receptor negative between the two groups, but other tumor and patient characteristics were similar in women with breast cancer in the dietary intervention and control groups. After 11.5 years of median follow-up, there were 516 deaths: 188 (37%) from breast cancer, 101 (20%) from other cancers, and 91 (18%) from cardiovascular disease (14). Women with breast cancer in the dietary intervention arm had a lower risk of all-cause mortality compared with women with breast cancer in the usual care group (HR = 0.78, 95% CI = 0.65 to 0.94). Risk of death from cardiovascular disease was lower (HR = 0.62, 95% CI = 0.39 to 0.99), and there was a trend toward lower risk of breast cancer-specific mortality (HR = 0.86, 95% CI = 0.64 to 1.17) in women with breast cancer in the dietary intervention group versus those with breast cancer in the control group.

In this issue of the Journal, Chlebowski and colleagues present additional information regarding mortality outcomes in women diagnosed with cancer during participation in the WHI MD Trial (15). During a median 17.7-year follow-up, 3867 deaths occurred in individuals diagnosed with cancer during study participation. As was reported in prior publications (13,14), there was a lower risk of all-cause mortality (HR = 0.85, 95% CI = 0.74 to 0.99) and a non-statistically significant trend toward a lower rate of breast cancer-specific mortality (HR = 0.87, 95% CI = 0.7 to 1.10) in women who developed breast cancer while participating in the low-fat dietary intervention versus the control group. However, there were no differences between the dietary intervention and control arms in cancer-specific or all-cause mortality in women who developed other types of cancer during study participation.

Why would the dietary intervention be related to mortality in women with breast cancer but not other cancers? One potential explanation may lie in the cause of death of women with breast cancer versus women with other cancers in the WHI DM trial. It is notable that only 10–11% of women diagnosed with breast cancer in the WHI DM trial died of the disease, with breast cancer accounting for 37% of deaths in these participants. Conversely a much greater proportion of the deaths that occurred after the diagnosis of other cancers were cancer related. Given the known benefits of dietary modification and modest weight loss on other health outcomes, and the significantly lower risk of cardiovascular deaths in women with breast cancer in the dietary intervention arm (14), it is possible that the effects of a low-fat diet on other health outcomes were more important in women in this trial who developed breast cancer given their relatively low risk of cancer-related mortality.

The authors raise another possible explanation for these differences in overall mortality in breast cancer patients: women randomized to the dietary intervention arm were less likely to develop cancers that were estrogen receptor positive and progesterone receptor (PR) negative as compared with patients in the control group (14.2% vs 18.8%, *P* = .5) (14). Given that PR negative cancers tend to be less responsive to endocrine therapy and more likely to recur (16), these differences in tumor subtype could also have affected mortality rates in the two groups. It is possible that these differences occurred as a result of the dietary intervention; a few other studies have suggested that body weight is associated with breast cancer tumor subtypes, with obese individuals being more likely to develop Luminal B tumors as opposed to Luminal A tumors (17,18). Regardless of the origin of the differences in PR positivity between the two groups, however, it makes it more difficult to conclude that participation in the dietary intervention after breast cancer diagnosis in these women led to improvements in mortality outcomes since the groups were not balanced at baseline. It could be that participation in such a lifestyle intervention is needed *before* cancer is diagnosed to influence tumor characteristics. These issues highlight the challenges in interpreting the effects of the WHI dietary intervention on outcomes of cancers that occurred during trial participation.

Should these new findings from the WHI DM trial affect the way we treat our patients? Two randomized trials have already addressed the impact of dietary modification on breast cancer recurrence and mortality in women with early-stage breast cancer, with mixed results. The Women Interventional Nutrition Study (19) and the Women’s Healthy Living and Eating Study (20) both enrolled women who had undergone surgery with or without chemotherapy and radiation for stage I–III breast cancer and randomized them to a low-fat dietary intervention versus control. Although both studies were successful in affecting dietary change in study participants, the Women Interventional Nutrition Study demonstrated an impact of the dietary intervention on breast cancer recurrence, with women randomized to the dietary intervention having a 24% reduction in the risk of breast cancer recurrence (19), but the Women’s Healthy Living and Eating Study did not demonstrate any impact of the dietary intervention on the risk of cancer recurrence or mortality (20).

The new results from the WHI DM continue to suggest that lifestyle factors may influence outcomes in women with early-stage breast cancer and provide provocative data that intervention after cancer diagnosis might improve survival. A number of on-going studies will directly test this hypothesis; the Breast Cancer Weight Loss (21) and SUCCESS C (22) trials will test the impact of caloric restriction and increased physical activity on cancer recurrence and morality in overweight and obese women with early-stage breast cancer, and the DIANA-5 trial (23) will test the impact of a macrobiotic, Mediterranean diet and exercise in women with breast cancer.

Although counseling women to consume a healthy diet after breast cancer diagnosis is certainly warranted for general health, the existing data still fall a bit short of proving this will help reduce the risk of breast cancer recurrence and mortality. On-going research will provide more definitive evidence regarding the potential benefits of lifestyle change after cancer diagnosis, evaluating not only the value of dietary modification but also of weight management and physical activity in women with breast cancer. These studies will hopefully provide oncologists with a more tailored prescription for lifestyle modification to optimize breast cancer outcomes.

## Notes

Affiliations of author: Division of Women’s Cancers, Dana-Farber Cancer Institute, Boston, MA (JAL).

The author has no disclosures to report.
